# Establishment and Preliminary Characterization of Three Astrocytic Cells Lines Obtained from Primary Rat Astrocytes by Sub-Cloning

**DOI:** 10.3390/genes11121502

**Published:** 2020-12-13

**Authors:** Fabio Caradonna, Gabriella Schiera, Carlo Maria Di Liegro, Vincenzo Vitale, Ilenia Cruciata, Tiziana Ferrara, Pietro D’Oca, Riccardo Mormino, Simona Maria Angela Rizzo, Italia Di Liegro

**Affiliations:** 1 Department of Biological, Chemical and Pharmaceutical Sciences and Technologies, University of Palermo, Viale delle Scienze, edificio 16, 90128 Palermo, Italy; fabio.caradonna@unipa.it (F.C.); gabriella.schiera@unipa.it (G.S.); carlomaria.diliegro@unipa.it (C.M.D.L.); vincenzo.vitale@hotmail.com (V.V.); ilenia.cruciata@unipa.it (I.C.); ferrarati@libero.it (T.F.); docapietro22@gmail.com (P.D.); riccardo.mormino@community.unipa.it (R.M.); simonamrizzo@gmail.com (S.M.A.R.); 2 Department of Biomedicine, Neurosciences and Advanced Diagnostics, University of Palermo, Via del Vespro, 129, 90127 Palermo, Italy

**Keywords:** gliomas, astrocytes, astrocyte cell lines, epigenetic alterations, linker histone H1.0

## Abstract

Gliomas are complex and heterogeneous tumors that originate from the glial cells of the brain. The malignant cells undergo deep modifications of their metabolism, and acquire the capacity to invade the brain parenchyma and to induce epigenetic modifications in the other brain cell types. In spite of the efforts made to define the pathology at the molecular level, and to set novel approaches to reach the infiltrating cells, gliomas are still fatal. In order to gain a better knowledge of the cellular events that accompany astrocyte transformation, we developed three increasingly transformed astrocyte cell lines, starting from primary rat cortical astrocytes, and analyzed them at the cytogenetic and epigenetic level. In parallel, we also studied the expression of the differentiation-related H1.0 linker histone variant to evaluate its possible modification in relation with transformation. We found that the most modified astrocytes (A-FC6) have epigenetic and chromosomal alterations typical of cancer, and that the other two clones (A-GS1 and A-VV5) have intermediate properties. Surprisingly, the differentiation-specific somatic histone H1.0 steadily increases from the normal astrocytes to the most transformed ones. As a whole, our results suggest that these three cell lines, together with the starting primary cells, constitute a potential model for studying glioma development.

## 1. Introduction

Brain cancers are complex and heterogeneous, and derive, in most cases, from glial cells [1,2,3,4,5]. These latter tumors are called gliomas, and are further subdivided into astrocytomas, oligodendrogliomas, ependymomas and glioastrocytomas [6,7]. In the last two decades, on the basis of next-generation sequencing, it has been realized that the different tumor types can differ for only a few gene mutations [8,9,10,11,12]. For example, grade IV glioblastoma, the most malignant glioma type, which accounts for 60–70% of gliomas, can be genetically subdivided into two main subtypes: isocitrate dehydrogenase (IDH)-wild type (or primary), and IDH-mutant (or secondary); the two subtypes are identical from the histo-pathological point of view, but show different clinical progression [5]. Cells with the IDH mutation overproduce 2-hydroxyglutarate (D-2HG), that inhibits α-ketoglutarate-dependent dioxygenases, including Jumonji (JmjC) domain-containing histone demethylases, and the ten-eleven translocation (TET) family of 5′-methlycytosine hydroxylases; this latter modification causes a genome-wide hypermethylation of CpG islands, which in turn silences various genes encoding cellular differentiation factors [12]. On the basis of these findings, it is now recommended to perform an “integrated diagnosis” of tumors, based on different criteria, such as histologic classification, the grade according to the World Health Organization (WHO), and molecular information [12].

Therapy of gliomas is mainly based on surgery, followed by radiotherapy and chemotherapy, aimed at limiting cell growth, and angiogenesis [13,14,15]. However, these approaches are not sufficient to reach all the infiltrating cells that can still invade the healthy brain tissue. In addition, long-term treatments with radiotherapy can induce cognitive impairment [16]. More recently, proton radiotherapy has been suggested to be less damaging than photon radiotherapy; however, its side effects are still to be clarified [17,18]. A further problem arises from development of drug resistance. Because of the many limits shown by the classical therapies, researchers are now looking for novel molecular approaches, allowing early diagnosis, on one hand, and more efficient treatments, on the other [19,20]. In particular, to understand the molecular details of the process that generates an invasive phenotype, and to find out novel biomarkers should be of the most importance in order to envisage new approaches to therapy.

H1.0 is a highly conserved histone variant belonging to the class of linker histones, and fundamental for the stabilization of mammalian chromatin higher order structures [21]. In contrast to the mRNAs encoding the other somatic linker histones (H1.1, H1.2, H1.3, H1.4, H1.5), H1.0 expression is replication-independent and the corresponding mRNA is poly-adenylated [22,23]. In rat embryos, H1.0 appears only in differentiated cells, while in actively proliferating tissues, such as thymus and spleen, it is kept at low levels [24,25], thus suggesting that its role could be the maintenance of the differentiated state [26]. More recently, it was reported that tumors are highly heterogeneous and that a decrease in H1.0 expression contributes to determine maintenance of long-term self-renewal potential [27,28]. Thus, H1.0 protein might be involved in the regulation of the “maintain pluripotency-or-differentiate” decision of the embryonic stem cells, also in the context of cancer growth [21]. Interestingly, at least some tumor cells do synthesize H1.0, but they seem to discard it through extracellular vesicles (EVs) [29,30]. H1.0 mRNA shows a long 3′-UTR, containing recognition sites for different RNA-binding proteins (RBPs) [31,32,33], including CSD-C2 (also known as PIPPin) [23,33,34]. These RBPs are probably involved in the post-transcriptional regulation of H1.0 histone synthesis during development and differentiation. In particular, CSDC2 has been reported to inhibit H1.0 mRNA translation [35]. Notably, this protein has been recently reported as one of the proteins with good prognostic value for patients with early-onset colorectal cancer [36]. In addition, H1.0 mRNA interaction with RBPs probably also controls its sorting to EVs [30].

When investigating cancer, and especially brain cancer, researchers need easily available in vitro models on which to study in detail the molecular events underlying transformation, in the absence of bioethical concerns. In order to obtain a cellular system suitable for analyzing the molecular determinants of astrocytic transformation, in the present study we selected three populations of cells with increased division rate: we started from primary astrocytes purified from fetal rat brain and selected, as described below, a first clone (A-GS1) with a higher rate of growth. By sub-cloning these cells, we obtained a second cell line (A-VV5) with a still higher rate of propagation. Finally, by sub-cloning A-VV5, we obtained a cell line (A-FC6) that still retained some astrocytic properties, including expression of the glial fibrillary acidic protein (GFAP), but hosting chromatin alterations typical of cancer cells. We analyzed all three cell lines at the cytogenetic and epigenetic levels, in comparison with normal primary astrocytes. In addition, we analyzed the ability of all the cells to produce the histone variant H1.0.

## 2. Materials and Methods

### 2.1. Purification of Primary Astrocytes from Cortices of Rat Fetuses

Normal astrocytes used as starting material were prepared from rat fetuses at the 16th day of gestation. For the present study we used the brain material left over in parallel experiments aimed at isolating neurons, thus avoiding any waste of brain tissue isolated from killed fetuses; such experiments were approved by the animal Welfare Committee of the University of Palermo and authorized by the Ministry of Health (Rome, Italy; authorization number 69636.N.GCQ). Briefly, cerebral hemispheres from 16-day-old rat fetuses were surgically removed, and placed in Dulbecco-Vogt modification of Eagle’s medium (DME) + 20% newborn calf serum (NCS). After removing the meningeal coverings, the tissue was fragmented by passing it through a Pasteur pipette and then dissociated by filtration through a nylon sieve (50 μm), while applying a gentle pressure with a rubber policeman. In order to remove aggregates and cerebral vessels, the cell suspension was then filtered through a second nylon sieve (25 μm). After centrifugation at 300× *g* for 5 min, cells were washed twice in DME, suspended in DME/Hams F-12 (2/1) supplemented with 10% heat-inactivated fetal calf serum (Sigma-Aldrich, St. Louis, MO, USA), and finally plated and cultured for a few days, up to confluence. In order to separate astrocytes from oligodendrocytes, we used a shaking procedure that takes advantage of the differences in adhesion of the various brain cell types, with astrocytes having a higher tendency to settle on the bottom of the culture flasks [37].

Some confluent astrocytes were detached with trypsin-EDTA, diluted in 93% fetal calf serum, 7% dimethyl sulfoxide (DMSO) and frozen. As already reported, indeed, astrocytes, frozen as soon as purified, can successfully recover and start proliferating, when thawed in fresh medium [38].

### 2.2. Establishment of Astrocyte Clones

In order to isolate new cell lines, characterized by increasing genomic, (epi)genomic and epigenetic instability, the limit dilution technique was used. This technique consists of performing serial dilutions of the progenitor cells, which are then cultured at a concentration ideally minor than 1 cell per well. The method is based on the concept that, in a given population, every cell can show a different level of genomic instability [39]. In detail, starting from primary astrocytes, we cultured the cells up to confluence, then diluted them down to approximately 1 cell/100 μL of medium, and cultured them in a 96-well plate (100 μL per well). All the wells were constantly examined in order to monitor the number of clones formed. At the end, one clone, growing at a visibly higher rate, was intentionally selected, the doubling time being a first important sign of genomic instability. The chosen clone was first transferred to a 24-well plate and then to a 6-well plate, and was named A-GS1. This procedure was repeated, following the same procedure, but using the A-GS1 as the starting cell line, and the A-VV5 clone was obtained. Finally, limit cell dilution of A-VV5 gave us the A-FC6.

### 2.3. Cytogenetic Analysis

In order to determine the cytogenetics differences in the three new cell lines, with respect to the standard karyotype of Norway rat (2*n* = 42) [40], they were cultured in triplicate in 75 cm^2^ flasks, as previously reported [41]. A suitable number of cells was initially seeded so that the culture never left the exponential growth phase. The doubling time of each cell line was obtained based on the number of metaphases obtained after treatment with colcemid (0.1 µg/mL). Colcemid treatment was performed 3 h before the trypsinization. The cultures were then processed according to the conventional air-drying protocol, as described previously [42,43]. Some of the slides obtained were used for standard Giemsa stain, others for G-banding [44], or for subsequential cytogenomic analyses. For each cell line, the modal karyotype was determined on 100 photographed metaphases, after standard Giemsa stain, while 30 G-banded metaphases of each cell line were examined to determine the presence of aneuploidies and/or structural aberrations [45].

### 2.4. Methylation-Sensitive Arbitrarily-Primed—Polymerase Chain Reaction (MeSAP-PCR)

The assessment of DNA Methylation at the genomic level was carried out, in three independent experiments, by using MeSAP-PCR as previously described [46,47] with some modifications. DNA extracted [48] from each cell culture was digested with RsaI restrictrion endonuclease (“single-digested DNA”: SDD). Then, half of the single-digested DNA was further treated with the methylation-sensitive restriction endonuclease HpaII, which is unable to cut DNA if a methylated cytosine is present in its recognition site (“double-digested DNA”: DDD). Single and double-digested DNA were separately amplified by AP-PCR with a 3′ GC-tailed arbitrary primer, capable to address amplification in CpG islands. The electrophoretic analysis of the amplicons obtained from SDD and DDD produced patterns that were different in terms of appearing/disappearing or attenuation/intensification of bands, as evaluated by a densitometry software. In general, the higher the variation in the pattern of bands, the higher the demethylation level in the analyzed genome.

### 2.5. In Situ 5-Methylcytosine Immunolocalization

With the aim to obtain a cytogenomic confirmation/visualization of the MeSAP-PCR results, we performed in situ chromosomal immune-localization [49] of the 5-methylcytosine (5-meC), using an anti-5-methylcytosine FITC-conjugate antibody on metaphase chromosomes of the astrocyte cell lines, as previously described [50]. Green brilliant grains indicate high concentration of 5-meC on a chromosome region, while a faint green fluorescence, that reveals the typical orange color of the propidium iodide counterstaining, is due to a poor methylation level.

### 2.6. Methylation-Sensitive Restriction Endonuclease Polymerase Chain Reaction (MSRE-PCR)

The evaluation of DNA methylation at the single gene level was carried out, in three independent experiments, by using Methylation-Sensitive Restriction Endonuclease Polymerase Chain Reaction (MSRE-PCR) [51]. PCR was carried out as previously reported [52,53], with a few modifications [54]. By using, as template, a DNA which was previously restricted by a methylation sensitive-restriction endonuclease, the appearance of a PCR product shows the presence of methylated CpG sites; on the contrary, the absence of an amplification band demonstrates the absence of site-specific methylation. By bioinformatics research and analysis, performed at the National Center of Biotechnology Information site (NCBI-https://pubmed.ncbi.nlm.nih.gov/) and by NEBcutter V2.0 software (http://tool.neb.com/NEBcutter2), five CpG sites were highlighted and selected in the promoter of the *H1F0* gene whose study was compatible with the MSRE-PCR technique and corresponding to the cleavage sites of the HpaII enzyme.

### 2.7. Preparation of Cell Lysates and Western Blot Analysis

Primary astrocytes as well as cells of the three clones were collected, washed with PBS, and homogenized in homogenization buffer (0.32 M sucrose; 50 mM sodium phosphate buffer, pH 6.5; 50 mM KCl, 0.5 mM spermine; 0.15 mM spermidine; 2 mM EDTA, and 0.15 mM EGTA), containing protease inhibitors (antipain, 2 μg/mL; aprotinin, 2 μg/mL; benzamidine, 1.0 mM; leupeptin, 2 μg/mL; pepstatin A, 2 μg/mL; phenylmethylsulfonyl fluoride, 1.0 mM, Sigma-Aldrich, St. Louis, MO, USA). Total protein concentration was determined by the Ouant-iT™ protein assay using a Qubit™ fluorometer (Invitrogen, Carlsbad, CA, USA). Equal amounts of proteins (10–20 μg) were loaded onto each lane of 12% polyacrylamide-SDS denaturing gels. After electrophoresis, samples were blotted onto PVDF membranes (0.45 μm pore-size, Amersham Biosciences, Little Chalfont, United Kingdom). Correct transfer of proteins to the membrane, and concentrations of the samples were visualized by staining with Ponceau red for 5 min. Finally, membranes were immunostained with a mouse monoclonal anti-H1.0 antibody (Santa Cruz Biotechnology, Santa Cruz, CA, USA). The secondary anti-mouse antibody was from Sigma-Aldrich (St. Louis, MO, USA).

### 2.8. Immunofluorescence Microscopy

Cells were cultured on coverslips for 48 h. Then, they were fixed with 96% ethanol, on ice, for 10 min and permeabilized for 5 min with 0.25% Triton X-100, in PBS. Cells were finally incubated with rabbit polyclonal anti-glial fibrillary acidic protein (GFAP) (Sigma-Aldrich, St. Louis, MO, USA). The secondary antibody was anti-rabbit-IgGs, conjugated to rodhamine (Sigma-Aldrich, St. Louis, MO, USA). Nuclear DNA was also stained with 4’-,6-diamino-2- phenylindole (DAPI; Vector Laboratories, Youngstown, OH, USA). The samples were finally observed in an Olympus BX-50 microscope (Olympus Italia s.r.l., Segrate, Italy) equipped with Vario Cam B/W camera (Nikon Instruments s.p.a., Calenzano, Italy).

### 2.9. Statistical Analysis

All the experiments were repeated at least three times. Western blots were scanned by the Image J program, and the values were normalized respect to the values obtained from the ponceau red staining of the same membrane, and/or respect to the values obtained by Coomassie blue staining of a portion of the membrane cut away before the Western analysis (the piece of membrane to be cut away contained proteins with a molecular mass higher than 50 kDa). After finding mean values and standard deviations, comparison between each couple of means (A-GS1 vs. PA; A-VV5 vs. PA, etc.) was done by the two-tailed Student’s *t*-test.

## 3. Results

### 3.1. Astrocytic Clones Maintain Expression of Glial Fibrillary Acidic Protein (GFAP)

**Figure 1:**
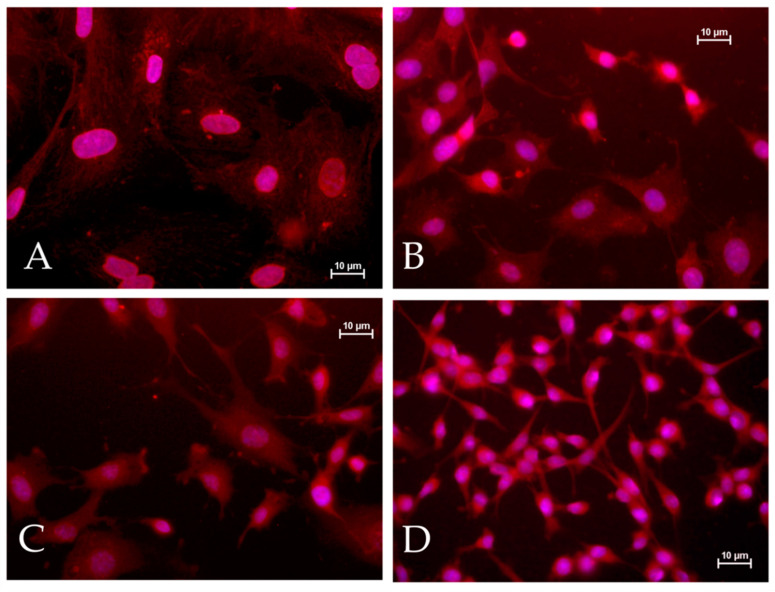
Immunofluorescent analysis of glial fibrillary acidic protein (GFAP) expression in primary astrocytes (**A**), A-GS1 clone (**B**), A-VV5 clone (**C**), and A-FC6 clone (**D**).

As demonstrated by immunofluorescence with anti-GFAP antibodies (Figure 1), both primary astrocytes and the clones derived from them express GFAP. However, morphological changes are clearly visible when comparing primary astrocytes (Figure 1A) with the clones (Figure 1B–D); in particular, cells of A-VV5 (Figure 1C) and, even more, cells of A-FC6 (Figure 1D) tend to be smaller and to have less extensions when compared with primary astrocytes that, on the other hand, tend to form a web of cells, still visible in cells of A-GS1 (Figure 1B).

### 3.2. Karyotype/Chromosome Instability Evaluated by Cytogenetic Data

The increasing instability of the cell lines was preliminarily demonstrated by their cell doubling time. This latter value was determined, for each cell population, by starting at the same time a series of different cultures, each of which was then blocked with colcemid at a different time. By knowing that colcemid has an effect only on mitotic cells, we measured the doubling time of that cell population (primary astrocytes or clone) as corresponding to the time at which we detected the maximal number of metaphases after colcemid treatment. We found that primary astrocytes and A-GS1 doubled in about 60 h; while A-VV5 and A-FC6 showed doubling times of 36 and 25 h, respectively. In other words, the cell duplication time became steadily shorter from primary astrocytes to the A-FC6 cells.

**Figure 2:**
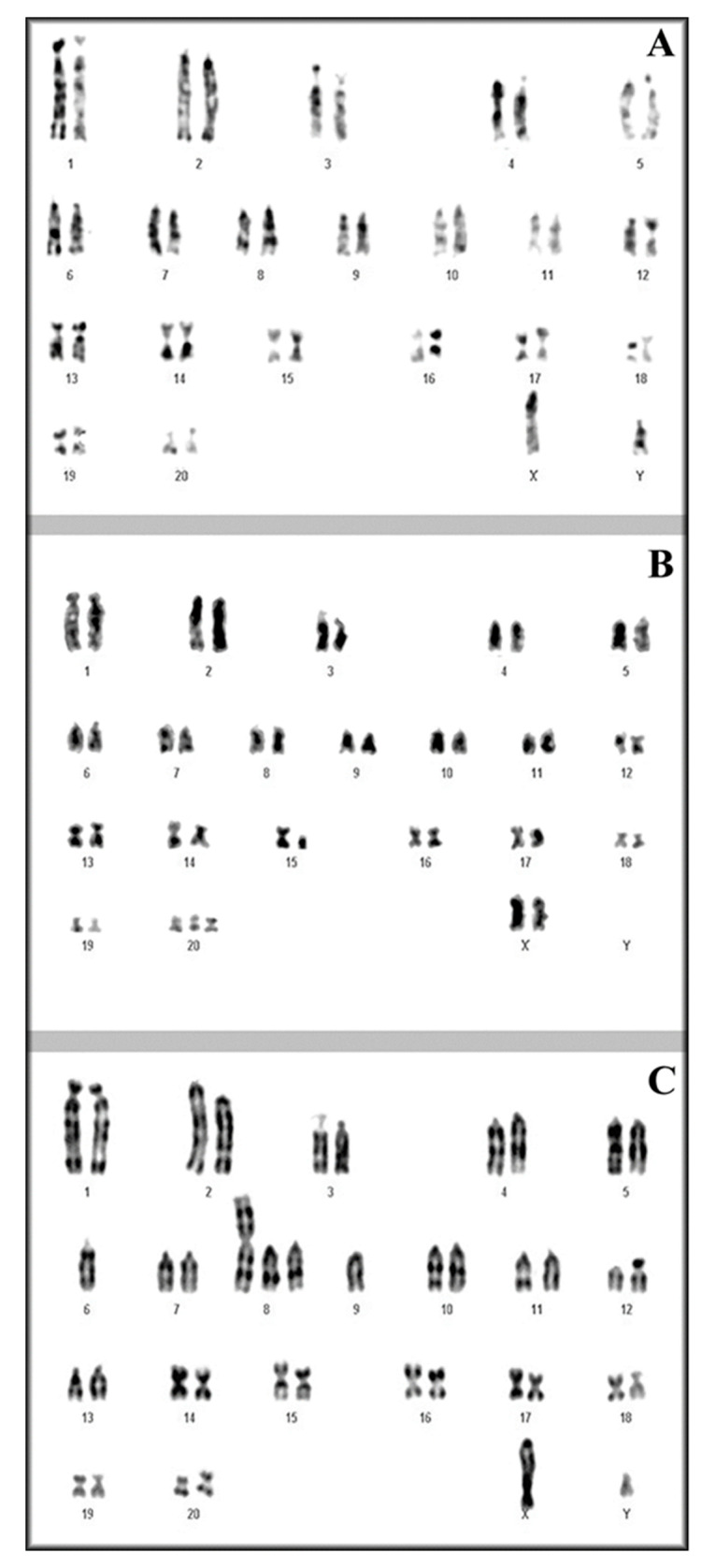
Representative karyotypes of (**A**) Primary astrocytes, (**B**) A-GS1, (**C**) A-FC6. In primary astrocytes no chromosome aberration is visible; in B, clonal aneuploidy was evidenced (e.g., trisomy #20); in C, both clonal aneuploidy and clonal chromosome aberration were present [e.g., monosomy #9 and i(8q)].

**Figure 3:**
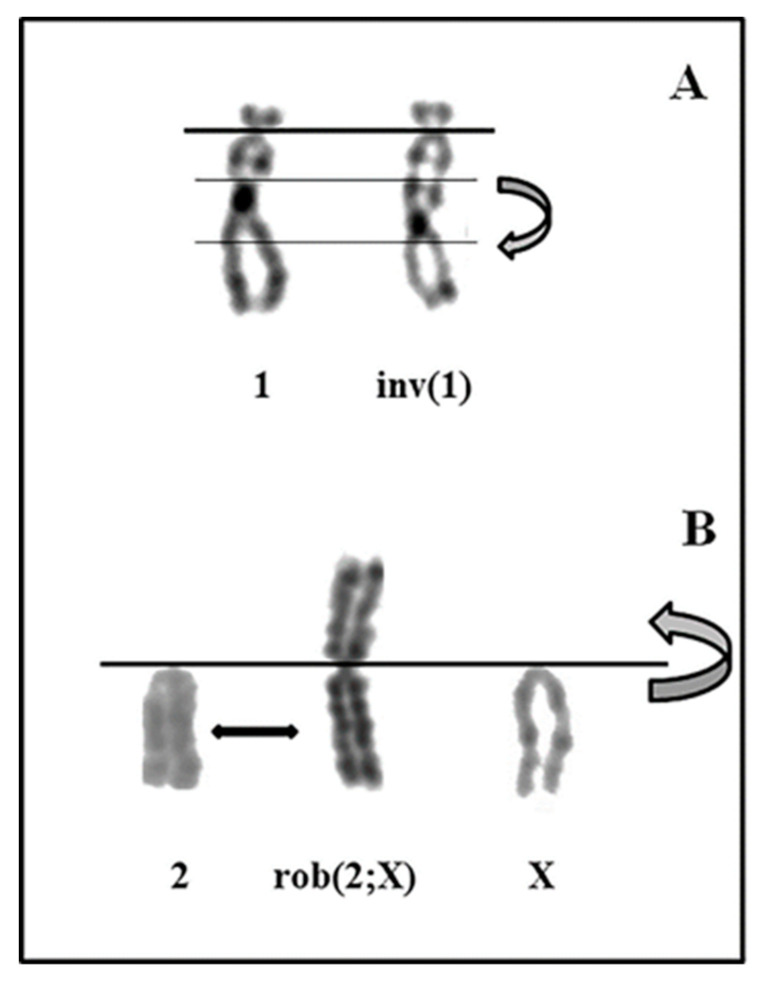
Partial karyotype with reconstruction of two clonal aberrations found in A-VV5. (**A**) Inversion of chromosome #1, (**B**) Robertsonian translocation between chromosomes #2 and #X.

**Figure 4:**
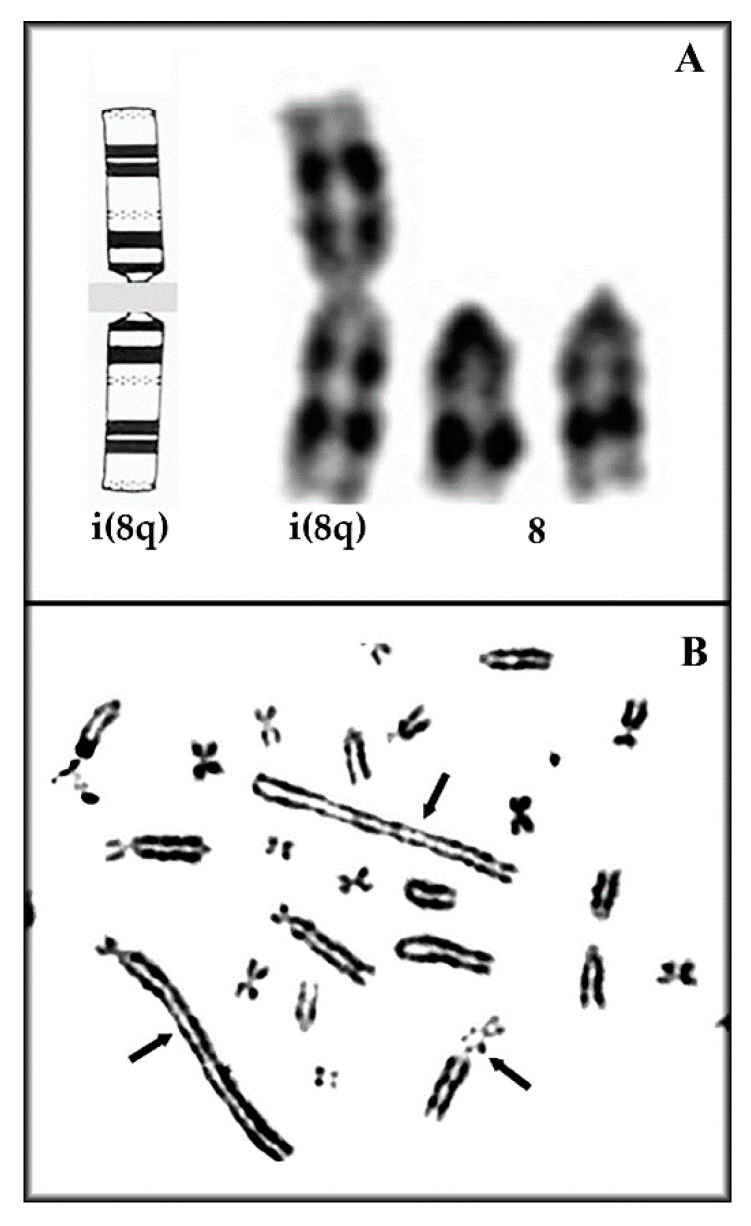
Representative clonal chromosome aberrations seen in A-FC6. (**A**) Ideogram reconstruction and isochromosome #8 seen in 100% of metaphases, (**B**) dicentric and triradial chromosomes (arrows).

The modal karyotypes were then determined by analyzing more than 100 metaphases and by selecting 30 good ones for each of the four cell lines. Genikon software (Nikon, Tokyo, Japan) was used for the karyotyping. As shown in Table 1, the percentage of standard euploid chromosome number (2*n* = 42) was found in a decreasing percentage from primary astrocytes to A-FC6 cells. Conversely but coeherently, all the remaining studied metaphases presented an abnormal number of chromosomes, showing an increasing percentage of aneuploidy (Figure 2). Regarding chromosome aberrations, Table 1 also demonstrates an increasing percentage of aberrations, dicentric chromosomes, and unidentified marker(s) up to the presence, in all the metaphases studied in the A-FC6 cells, of a stable additional i(8q) chromosome (Figure 3 and Figure 4).

### 3.3. Decrementation of Epigenomic DNA Methylation Revealed by MeSAP-PCR

**Figure 5:**
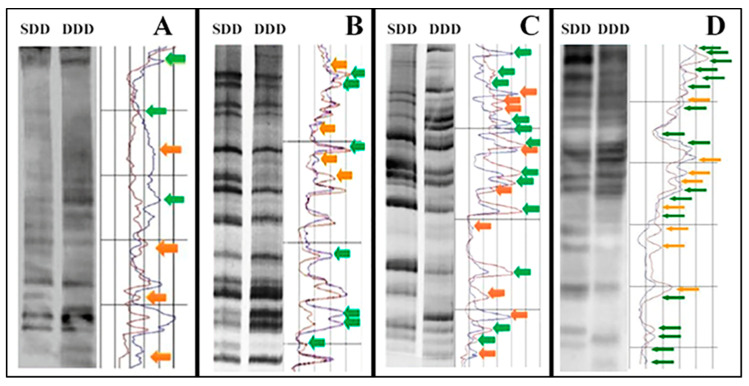
MeSAP DNA-Fingerprinting and relative densitometer scanning of primary astrocytes (**A**) and clones (**B**–**D**). The yellow arrows indicate appearing/disappearing and the green ones indicate attenuation/intensification of bands. By analyzing band pattern differences between single-digested DNA (SDD) and double-digested DNA (DDD) it is possible to evidence 7 variations in primary astrocytes (**A**), 11 in A-GS1(**B**), 21 in A-VV5 (**C**) and 25 in A-FC6 (**D**).

DNA fingerprinting, analyzed by densitometer scanning software, showed an increase in banding pattern differences between SDD and DDD, in terms of both appearing/disappearing and attenuation/intensification of bands (Figure 5). These findings demostrate a decrease in DNA methylation from primary astrocytes to A-FC6 clone. In particular, only seven differences were found in primary astrocytes, while 11 were visible in A-GS1, 21 in A-VV5 and 25 in A-FC6.

### 3.4. Cytogenomic Confirmation of DNA Demethylation by In Situ 5-Methylcytosine Immunolocalization

**Figure 6:**
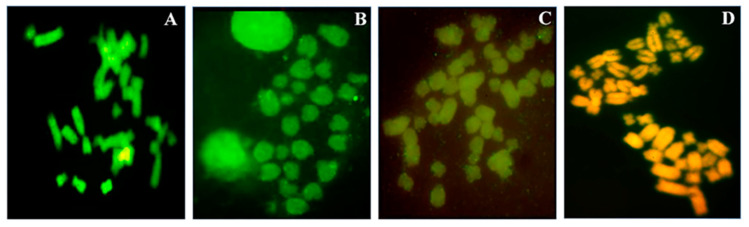
Chromosomal 5-methylcytosine indirect immunolocalization on metaphases of primary astrocytes and clones by anti-5-methylcytosine FITC-conjugate antibody. Representative metaphases of primary astrocytes (**A**), A-GS1 (**B**), A-VV5 (**C**) and A-FC6 (**D**). Green brilliant grains indicate high concentration of 5-meC, while a faint green fluorescence is due to a low level of methylation; when the green fluorescence is very low, the typical orange color of the propidium iodide counterstaining becomes predominant (**D**).

Metaphases, indirectly immunostained to reveal in-situ chromosomal 5-methylcytosine, showed a steady decrease in chromosomal 5-methylcytosine from primary astrocytes to A-FC6. As clearly shown in Figure 6, metaphases of primary astrocytes were intensely green, with brilliant grains, while those of A-GS1 were green, but without brilliant grains. Metaphases of A-VV5 were faint-green and, lastly, those of A-FC6 showed no green chromosomes, making evident only the normal chromatin colour.

### 3.5. Epigenetic Promoter Methylation Signatures Displayed by MSRE-PCR

We also evaluated methylation in the promoter of the gene encoding H1.0 (*H1F0*). In particular, we analyzed five valuable *CG* sites (Table 2). As shown in the table, no difference in the methylation pattern was found.

### 3.6. Western Analysis of H1.0 Expression

**Figure 7:**
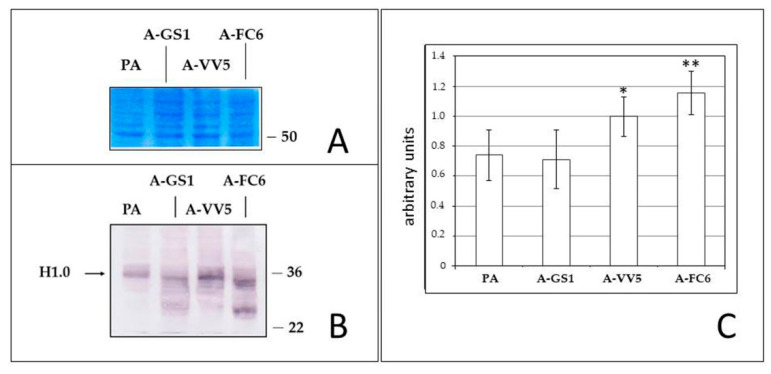
Western blot analysis of total lysates from primary astrocytes (PA), and from the three clones derived from them. **(A)** Coomassie blue staining of a piece of the same membrane used for the western blot analysis shown in (B); (**B**) representative blot showing distribution of the bands recognized by the anti-H1.0 antibody; position of the main expected H1.0 band is indicated, together with the positions of two reference markers; (**C**) graphical representation of the statistical analysis. Only the main upper band was considered. * *p* < 0.01 (vs. PA); ** *p* < 0.001 (vs. PA).

Since the gene encoding H1.0 is methylated at the same level in primary astrocytes and in the clones derived from them, we also analyzed the expression of the H1.0 protein, also because it was reported that its expression in the developing rat brain is mainly regulated at the post-transcriptional level [55]. As shown in Figure 7, H1.0 is present in the total cell lysates of all the cell populations analyzed, with a tendency of the main, expected band to increase from primary astrocytes (PA, in the Figure) to the most transformed A-FC6 clone. Moreover, faster migrating bands of still unknown origin are recognized by the antibody in the clones, and especially in A-FC6 cells.

## 4. Discussion

Gliomas, or at least some subtypes, are considered among the deadliest cancers. Extensive research at the cellular and molecular level could provide a better view of brain cancer from a biological point of view, useful for developing and evaluating new therapeutic approaches. However, in vivo animal models are complex, expensive, and require care and personnel for a long time. For these reasons, in vitro models of gliomas can be of the most importance for molecular analyses. For example, in vitro models have been described for the interaction between malignant glioblastoma cells and the extracellular matrix [56], which unfortunately do not return information on the genetic, epigenomic and epigenetic phenomena responsible for the initial phases of the neoplastic transformation of a brain cell. It is worth noting that, in a laboratory cell line, it is possible to study a phenomenon as an isolated fact, highlighting it from the convergence of the so-called disturbing factors. Thus, availability of a suitable in vitro system could represent an excellent model for investigating brain cancer evolution.

Distinctive hallmarks of cancer include increased proliferative activity, evading growth suppressors, resistance to cell death, induction of angiogenesis, and activation of metastasis [57,58,59]. These properties depend on the instability of the genome, which accelerates the acquisition of these characteristics through initial mutations and chromosomal rearrangements, which can lead to further aberrations and to acquisition of an overt transformed phenotype.

Starting from the concept that non-human cell lines can facilitate the study of genomic and epigenetic phenomena that drive a normal cell to acquire a tumor phenotype, we tried to select transformed astrocytic cell lines, and obtained three clones with evident traits of increasing genomic instability, as demonstrated by different approaches. It is widely accepted and has been recently confirmed that a short cell duplication time in solid tumors is associated with a poor prognosis [60]. The cells produced in the present study have shown a decreasing doubling time from the primary astrocytes to the A-FC6 clone, that shows the shortest cell cycle, of about 25 h. In addition, as clearly shown in Table 1 and in Figure 1 and Figure 2, both numerical and structural chromosomal abnormalities sequentially increase in the three clones obtained from primary astrocytes, in which abnormalities were not observed.

Genomic instability can be caused by chromosomal rearrangements, altered metabolism, oxidative stress [61], DNA-repair defects [62], and intrinsic alterations of specific genes [63]. In particular, in a population of unstable cells, specific chromosome rearrangements can be selected because of the cell propagation advantage thus obtained, due to duplication of oncogenes and/or loss of oncosuppressor ones. These latter conditions can be simultaneously present in cells with isochromosomes, a cancer-peculiar cytogenetic aberration in which a pair of identical arms are duplicated in a metacentric chromosome, that now contains a doubled supply of some genes, while many others are completely lost [64]. A-FC6 showed a high degree of aneuploidy (59%) and the presence, in 100% of the observed metaphases, of an isochromosome (iso (8)), due to duplication of the long arm of chromosome 8 (Figure 3A). This event leads to doubling of the genes present in the long arm of the chromosome. Notably, two genes involved in cancer map in *Rattus norvegicus* 8q: *IL8* and *ETS1*. IL-8 induces an increase in the expression of Matrix Metalloprotease (MMP) 13, a zinc-binding proteolytic enzyme that facilitates cell migration and progression of malignant glioma, also by increasing the blood brain barrier permeability [65]. Moreover, IL-8 plasma levels are commonly used as a prognostic marker of decreased survival in many malignant tumors [66]. On the other hand, ETS Proto-Oncogene 1 (*ETS1*) is expressed in different cancer subtypes and has been associated with the degree of glioma cell invasiveness [67]. Thus, unstable cells such as A-FC6, endowed with a double dose of these genes, can represent a cell population with a high degree of malignant and metastatic properties.

Epigenomic analyses also showed a constant increase in global DNA hypomethylation (Figure 5) among the four cell populations; cytogenomic immunolocalization of 5-meC (Figure 6) visually confirmed on chromosomes the data obtained by the semi-quantitative molecular techniques applied to DNA. It is a widespread observation that genomic DNA in cancer cells tends to be globally hypomethylated, and locally hypermethylated [50,68]. According to this paradigm, primary astrocytes show a high global DNA methylation, while, at the other extreme, A-FC6 cells, with their widespread demethylation of genomic DNA, can be considered as a cell line with a high degree of instability. Consistently, the other two clones showed an intermediate level of global DNA methylation. On the other hand, through epigenetic analysis, we found that the promoter of the *H1F0* gene, that encodes the linker histone H1.0, was methylated in all but one the analyzed CpG sites, in all the four cell populations, including the A-FC6 clone (Table 2). This observation might have suggested silencing of the gene, as expected for a gene encoding a protein that normally does not accumulate in proliferating and undifferentiated cells [21]: however, Western analysis showed that H1.0 protein is highly expressed in all the four cell populations, with even a tendency to increase in the A-FC6 clone. Actually, this finding was not completely unexpected, since it was previously found that both oligodendroglioma—and melanoma—cell lines synthesize high amounts of H1.0, but discard it into the extracellular environment through extracellular vesicles [29,30]. Interestingly, in addition to an increase in the main H1.0 band, other faster migrating bands are visible in the clones, and especially in A-FC6 cells. It will be of interest to study these forms in order to understand from which kind of biochemical modifications they derive and which relevance they may have in the transformation process.

## 5. Conclusions

By integrating the results obtained in the present study, we can conclude that the four cell populations selected and analyzed can be ideally placed on a path that goes from normal to malignant cells, with growing genomic instability. Even if the “in vitro/in vivo” extrapolation sometimes represents a weak point in cancer research [69], and although further methylomic and transcriptomic analyses are necessary to better characterize the three cell lines, based on the data till now collected, we believe that the non-human in vitro model described is potentially useful for studying glioma development.

## Figures and Tables

**Table 1:** Cytogenetic results found in the four cell lines.

Cell Line	Modal Karyotype (2*n*=)	Euploidy (%)	Aneuploidy (%)	of Which (Specific Aneuploidy)	Chromosome Aberrations (%)	of Which (Specific Aberration)
Primary astrocytes	42	100	0	-	0	-
A-GS1	43	59	41	➢ Trisomy #20 ➢ NCAWLCs ** ➢ NCAWGCs ***	0	-
A-VV5	41	45	55	➢ Monosomy #3 ➢ NCAWLCs ** ➢ NCAWGCs ***	11 7 2	➢ rob(2;X) ➢ inv(1) ➢ uCMARs *
A-FC6	41	41	59	➢ Monosomy #6 ➢ Monosomy #9	100 12 5	➢ i(8q) ➢ dicentric chrs ➢ uCMARs *

* uCMARs = Unidentified Chromosome Markers; ** NCAWLCs non-clonal aneuploidies with loss of chromosomes; *** NCAWGCs non-clonal aneuploidies with gain of chromosomes.

**Table 2:** Promoter CpG methylation of *H1F0*, in the four cell populations.

Gene →	*H1F0*				
CpG Site * →	844	1592	2352	3036	3586
Primary astrocytes	M	uM	M	M	M
A-GS1 clone	M	uM	M	M	M
A-VV5 clone	M	uM	M	M	M
A-FC6 clone	M	uM	M	M	M

M = Methylated; uM = unmethylated. * numeration corresponds to nucleotide position of HpaII (methylation sensitive restriction endonuclease) cutting site.
